# Development and validation of a prognostic risk model for pediatric patients with left-to-right shunt congenital heart disease and heart failure

**DOI:** 10.3389/fpubh.2025.1692007

**Published:** 2025-11-06

**Authors:** Li Mei Zhang, Yan Yun Huang, Yu Qin Huang, Yu Sheng Pang

**Affiliations:** Department of Pediatrics, The First Affiliated Hospital of Guangxi Medical University, Nanning, China

**Keywords:** left-to-right shunt congenital heart disease, heart failure, pediatrics, prognostic prediction, risk model, survival analysis

## Abstract

This retrospective study aimed to develop a reliable prognostic model for pediatric patients with left-to-right (L-R) shunt congenital heart disease (CHD) complicated by heart failure (HF), based on clinical data collected from 407 cases between August 2012 and June 2024. The cohort included 63.4% male patients with a median age of 4.33 months, and participants were randomly assigned to a training set (*n* = 284, 69.8%) and an internal validation set (*n* = 123, 30.2%). Univariate and multivariate Cox regression analyses combined with LASSO regression were used to identify key independent prognostic variables for overall survival (OS): modified Ross classification, N-terminal pro-brain natriuretic peptide (NT-proBNP) levels exceeding 5,743 pg./mL, elevated blood urea nitrogen (BUN), presence of shock, and history of thoracotomy. These factors were integrated to construct a nomogram, which showed strong prognostic performance—concordance indices (C-index) were 0.829 in the training set and 0.850 in the validation set. Survival analysis revealed significant differences in 3-, 6-, and 12-month OS between high-risk and low-risk groups stratified by nomogram scores. These findings suggest that the proposed nomogram, when applied to pediatric populations with L-R shunt CHD and HF who share baseline characteristics similar to those in our study, could serve as a promising auxiliary tool for early risk stratification and clinical decision-making.

## 1 Introduction

The estimated global mean prevalence of congenital heart disease (CHD) among live births was 8.2 per 1,000 from 1970 to 2017 (1). The most prevalent congenital heart defects are left-to-right (L-R) shunts, which include atrial septal defect (ASD), ventricular septal defect (VSD), patent ductus arteriosus (PDA), atrioventricular septal defect, and partial anomalous pulmonary venous connection. Occasionally, persistent arterial trunks, coronary artery right ventricular fistula, and main pulmonary artery window can also be observed (2, 3).

CHD is the primary etiology of heart failure (HF) in pediatric patients and a leading cause of death in children under five in China, particularly within the 0–1 age group (4, 5). Without timely, accurate, and effective intervention, mild cases may lead to physical development retardation and impaired nervous system development, while patients with severe CHD malformations face a significantly elevated risk of mortality.

Although the mortality rate associated with L-R shunt CHD is lower than that of complex CHD, L-R shunts account for a substantial portion of congenital heart defects, and comorbid HF further exacerbates mortality risk in this subgroup (6). Several factors—including age, primary diseases, complications, therapeutic methods, and levels of N-terminal pro-brain natriuretic peptide (NT-proBNP)—significantly influence the prognosis of pediatric HF (7, 8). However, there remains a dearth of uniform and quantitative prognostic tools for L-R shunt CHD complicated by HF, and existing predictive approaches have notable limitations that hinder their clinical utility for this specific pediatric population (9).

For instance, traditional risk scoring systems (e.g., unweighted summation of risk factors) treat all prognostic variables as equally impactful. They fail to account for the differential contribution of each factor. This oversimplification often leads to inaccurate risk stratification (10). It is especially problematic in heterogeneous groups, such as pediatric CHD patients with varying defect types and HF severity (11).

Single-biomarker or single-clinical-index models (e.g., using NT-proBNP alone to predict survival) overlook the multifactorial nature of HF prognosis in this population. They ignore critical confounders like renal function or hemodynamic instability. These confounders are common in pediatric L-R shunt CHD and directly affect outcomes (12, 13). As a result, these models lack comprehensiveness and tend to over- or under-estimate risk.

Machine learning-based predictive models can capture complex interactions between variables. But they are often black-box models. When machine learning models perform well and their predictions are explained to clinicians, they can be well adopted. Significant efforts have been made in this direction since Carvalho et al. 2019 (14).

Nomograms address these key limitations. They are uniquely suited for prognostic assessment in pediatric L-R shunt CHD with HF. First, nomograms integrate multiple predictive variables. They also quantify the weighted contributions of these variables based on statistical evidence. This ensures high-impact factors drive risk estimates, which aligns with the heterogeneous prognostic patterns of this patient group. Second, nomograms are visually intuitive. Clinicians can map a patient’s specific data to a total risk score. They can then directly read 3-, 6-, or 12-month survival probabilities, with no need for complex calculations. Third, unlike black-box models, nomograms offer full transparency. The contribution of each variable to the final risk is clear. This enhances clinician confidence and helps with patient-family communication. Finally, nomograms can be rigorously validated. Metrics like concordance index (C-index) and calibration curves are used for validation. This ensures their reliability across different cohorts.

Therefore, this retrospective cohort study aimed to develop a prognostic nomogram for predicting overall survival (OS) in pediatric patients with L-R shunt CHD and HF. By incorporating baseline characteristics and key clinical parameters, the model intends to provide a robust, precise tool for individualized prognosis assessment—ultimately enhancing risk stratification, facilitating early clinical intervention, supporting personalized treatment strategies, and contributing to optimized resource allocation and improved long-term outcomes in this high-risk population.

## 2 Methods

### 2.1 Study population

From August 2012 to June 2024, this study enrolled 407 participants from the First Affiliated Hospital of Guangxi Medical University and were randomly assigned to the training set (*n* = 284, 69.8%) and internal validation set (*n* = 123, 30.2%) by computer-generated random numbers.

The HF severity was assessed by employing the modified Ross heart failure classification for children (Supplemental File Supplementary Table S1). All subjects presented with L-R shunt congenital heart defects (Supplementary Table S2). The inclusion criteria for patients were as follows: (1) age less than or equal to 3 years; (2) CHD characterized by L-R shunts diagnosed by echocardiography; (3) HF was diagnosed based on the revised 2020 recommendations for the diagnosis and treatment of heart failure in children proposed by the Subspecialty Group of Cardiology, Society of Pediatrics, Chinese Medical Association (15). Additionally, a modified Ross score of 3 or higher was required for all patients; (4) Patients received either conventional anti-HF therapy or a combination of conventional anti-HF therapy and thoracotomy (16). Thoracotomy specifically refers to corrective open-heart surgery for L-R shunt CHD (e.g., ASD repair, VSD closure), excluding palliative procedures such as pulmonary artery banding; (5) Individuals whose legal guardians gave informed consent. Exclusion criteria: (1) premature infants with PDA, except for the preterm infants who required arterial ligation due to HF during hospitalization; (2) minor cardiac anomalies: patent foramen ovale, small isolated atrial septal defects (ASDs) (< 5 mm in diameter), small isolated ventricular septal defects (VSDs) (< 5 mm in diameter), or small isolated patent ductus arteriosus (PDAs) (< 3 mm in diameter); (3) patients with CHD who had previously undergone cardiac thoracotomy prior to admission; (4) patients with CHD complicated by malignant neoplasms, severe central nervous system diseases, or other heart diseases (such as cardiomyopathy, myocarditis, primary arrhythmia, infective endocarditis, the acute phase of Kawasaki disease, and idiopathic pulmonary hypertension); (5) Individuals who were not followed up or died from causes unrelated to HF; (6) participants exhibiting incompleteness, apparent inconsistencies, or inaccuracies in their clinical records. This study protocol was approved by the institutional research committee of our hospital.

### 2.2 Variables collection and follow-up

Variables used in this investigation included age, gender, race, premature delivery, chromosome aberration, body mass index, systolic/diastolic blood pressure, heart rate, modified Ross classification, echocardiographic data, laboratory data, cardiac and non-cardiac comorbidities after HF diagnosis as well as provision of thoracotomy and mechanical ventilation support. In the study, comorbidities were diagnosed based on relevant references (17–20) and evaluated by several attending physicians, including septic or cardiogenic shock, malignant arrhythmia, multiple organ dysfunction syndrome and pulmonary hypertensive crisis. Arrhythmias diagnosed by electrocardiogram or Holter monitoring and treated with antiarrhythmic medications were categorized as malignant, including atrial tachycardia, atrial flutter, atrial fibrillation, ventricular tachycardia, supraventricular tachycardia, second-degree type II atrioventricular block, and high-grade atrioventricular block. A total of 112 patients in this cohort underwent Holter monitoring.

It is worth emphasizing that 102 children with preoperative symptoms and signs of HF underwent thoracotomy after successful management of their cardiac insufficiency. The surgical intervention was performed by the pediatric cardiac surgery team at our hospital under the meticulous supervision of the anesthesia-cardiopulmonary bypass specialist team. After fulfilling the discharge requirements and obtaining physician consent, they were then followed up through telephone calls and regular outpatient visits at 3, 6, and 12 months after discharge. Notably, the follow-up duration of this study starts from the “HF diagnosis time” rather than “admission time” or “discharge time,” and the end point is defined as “occurrence of death” or “completion of 12-month follow-up.” This definition ensures that the calculation of survival time is directly associated with HF-related prognosis. For children discharged shortly after HF diagnosis, the follow-up start time still takes the diagnosis date as the reference, so as to avoid time bias caused by differences in hospital stay duration. The survival time was determined as the duration commencing at the point of HF diagnosis and extending until either death or the termination of the pre-established final follow-up period, and overall survival (OS) was meticulously documented.

To ensure the reliability of survival analysis, the completeness of follow-up data was systematically evaluated, with a focus on the acquisition of full 12-month follow-up information for all enrolled patients. Among the 407 pediatric patients included in the study, 392 (96.3%) completed the full 12-month follow-up schedule consistent with the aforementioned telephone and outpatient follow-up protocol. The remaining 15 patients (3.7%) had incomplete 12-month follow-up data: 8 patients were lost to follow-up (1.98%) due to changes in contact information or relocation to other regions, and 7 patients (1.72%) had follow-up terminated early because they died before reaching the 12-month time point (all deaths were related to HF, in line with the study’s outcome definition). When analyzed by risk groups (based on a nomogram score cutoff of 153 points), the follow-up completion rate was 97.1% (269/277) in the low-risk group (*n* = 277) and 94.6% (123/130) in the high-risk group (*n* = 130). There was no statistically significant difference in completeness between the two groups (χ^2^ = 1.236, *p* = 0.266), which ruled out the interference of follow-up completeness on risk stratification results.

For patients with incomplete follow-up, survival time was censored at the last effective follow-up time point (e.g., if a patient died at 5 months post-diagnosis, their survival time was recorded as 5 months, and no further follow-up data were required for the 12-month time point). Additionally, to minimize bias from lost follow-up, a sensitivity analysis was conducted: the survival curves of patients with complete 12-month data and those with censored data were compared using the log-rank test, and no significant difference was observed (*p* = 0.623), confirming that lost follow-up had no substantial impact on the study’s overall survival analysis results.

### 2.3 Statistical analysis

The sample size was derived from available data, and missing values were imputed using multiple imputations. Statistical analyses were performed with R 4.3.3. For continuous variables with a normal distribution were expressed as the mean ± standard deviation and t-tests were used to compare groups, and those with a nonnormal distribution, we utilized the median and interquartile range instead and performed Mann–Whitney U tests for group comparisons. Categorical variables are expressed as numbers and percentages, and chi-squared tests or Fisher’s exact tests were used to compare the distribution between the training set and validation set. Both the least absolute shrinkage and selection operator (LASSO) algorithm (The glmnet R package) and multivariate Cox regression to were used to ascertain predictive factors and model building, and we used Cox regression analyses to estimate the hazard ratios (HR) and their 95% CIs.

During the variable selection phase, potential confounding factors were systematically screened. The following variables were ultimately excluded from the final model, with reasons detailed below.

#### 2.3.1 Concurrent infections

Cases with infection-related deaths were excluded via “Exclusion Criterion 5” during enrollment. In multivariate analysis, concurrent pneumonia (*n* = 89) showed no statistically significant impact on OS (HR = 1.213, 95% CI: 0.735–1.998, *p* = 0.448), so it was not included in the final model.

#### 2.3.2 Nutritional status

Nutritional status was assessed using the Weight-for-Age Z-score (WAZ). Univariate analysis indicated that severe malnutrition (WAZ < −2) was associated with prognosis (HR = 1.876, *p* = 0.032). However, when included in the multivariate model, it exhibited collinearity with the “modified Ross classification” (Variance Inflation Factor, VIF = 3.82 > 3), and the adjusted HR was no longer statistically significant (HR = 1.325, 95% CI: 0.789–2.227, *p* = 0.289), leading to its exclusion.

#### 2.3.3 Pharmacotherapeutic regimens

A total of 92.1% (375/407) of patients in this study received standardized anti-HF treatment (diuretics + ACEI/ARB, with some combined with *β*-blockers), resulting in minimal variation in pharmacotherapeutic regimens. Univariate analysis showed no significant effect of different drug combinations on prognosis (*p* = 0.615), so this variable was not included in the model.

Before evaluating the model’s predictive performance, the proportional hazards (PH) assumption of the Cox regression model was verified using scaled Schoenfeld residual analysis. First, based on the multivariate Cox regression model constructed from the training set, scaled Schoenfeld residuals were calculated for each independent prognostic variable included in the model (i.e., modified Ross classification, NT-proBNP > 5,743 pg./mL, elevated BUN, presence of shock, and history of thoracotomy). These residuals quantify the discrepancy between the observed event times and the expected residuals under the assumption of constant HR over time, directly reflecting whether the effect of each variable changes with follow-up duration. Next, a graphical verification was performed. A roughly horizontal locally weighted regression curve indicated that the HR of the corresponding variable remained constant throughout the 3-, 6-, and 12-month follow-up periods, satisfying the PH assumption; in contrast, an obviously sloped curve suggested a time-dependent change in the variable’s effect, violating the assumption. To further quantify the verification results, linear regression analysis was conducted between the scaled Schoenfeld residuals of each variable and follow-up time. A non-significant regression coefficient (*p* > 0.05) confirmed no significant association between residuals and time, providing statistical evidence for compliance with the PH assumption. If any variable was found to violate the PH assumption, the variable was converted into a time-dependent covariate using the survSplit() function in R or a stratified Cox model was constructed by time strata to adjust for the violation, ensuring the reliability of the final prognostic model.

The model’s prediction accuracy was evaluated using the discrimination measured by the Harrell’s concordance index and its 95% CI. The model’s calibration was assessed through the calibration plots at 3,6 and 12 months. The discriminative capability was also evaluated using a time-dependent receiver operating characteristic (ROC) curve by the risk Regression package in R. Additionally, the assessment of its clinical application value was performed by constructing a clinical decision curve.

The nomogram model was utilized to calculate the nomogram score for all participants. In the training set, an optimal cutoff point for the nomogram score for the model was computed to stratify participants into low-risk or high-risk groups. The optimal cutoff was determined using log-rank statistics to provide the largest discrepancy in overall survival between the risk groups (surv_cutpoint function; survminer package in R). Subsequently, Kaplan–Meier survival curves were generated. The log-rank test was employed to compare OS between high-risk and low-risk groups at various time points. The statistical tests were two-tailed with a significance level of *p* < 0.05.

To further explore the clinical relevance of the risk stratification, additional analyses were conducted to assess the impact of surgical timing and quantify the therapeutic benefit of thoracotomy within each risk group. Specifically, based on the established nomogram score cutoff of 153 points (high-risk: score ≥153; low-risk: score <153), surgical timing was categorized into three groups using the interval between “heart failure (HF) diagnosis date” and “thoracotomy date”: ① early surgery (<1 week), ② intermediate surgery (1–2 weeks), and ③ delayed surgery (>2 weeks).

The Kaplan–Meier method was used to calculate 12-month survival rates for each subgroup stratified by both risk level and surgical timing, and the log-rank test was applied to compare survival differences among these subgroups. Furthermore, to quantify the mortality risk difference between surgical and non-surgical patients within the high-risk and low-risk groups separately, a multivariate Cox regression model was employed—with adjustments for potential confounders including age, modified Ross classification, and presence of shock. This model was used to estimate the hazard ratio (HR) and its 95% confidence interval (95% CI) for mortality. All statistical tests remained two-tailed with a significance level of *α* = 0.05.

## 3 Results

### 3.1 Patient characteristics and survival

The general clinical characteristics of 407 pediatric patients are shown (Table 1). The results indicated that, except for the RDWCV, there were no statistically significant differences observed in the remaining variables between the training and validation sets (*p* > 0.05). Out of a sample size of 407 individuals, with 63.4% male (*n* = 258) and 36.6% female (*n* = 149). The median age was 4.33 months, ranging from one day to three years, with 383 (94.1%) being less than or equal to one year old. Regarding age distribution, further stratification showed 16.7% (68/407) of patients aged <1 month, 55.3% (225/407) aged 1–6 months, 22.1% (90/407) aged 7–12 months, and 5.9% (24/407) aged 1–3 years; no significant difference in age stratification was observed between the training and validation sets (all *p* > 0.05).

**Table 1:** Baseline characteristics of study populations.

Variables	ALL (*n* = 407)	Training (*n* = 284)	Validation (*n* = 123)	*P*-value
Age (months)	4.33 (2.40, 6.38)	4.60 (2.82, 6.41)	3.97 (1.64, 6.31)	0.175
Male gender (*n*, %)				0.649
Race (*n*, %)	258 (63.4)	178 (62.7)	80 (65.0)	
Han	149 (36.6)	106 (37.3)	43 (35.0)	
Zhuang				0.073
Others	250 (61.4)	170 (59.9)	80 (65.0)	
Premature delivery (*n*, %)	68 (16.7)	48 (16.9)	20 (16.3)	0.873
Chromosome aberration (*n*, %)	41 (10.1)	27 (9.5)	14 (11.4)	0.564
BMI (kg/m^2^)	13.17 (11.94, 14.49)	13.10 (11.93, 14.42)	13.22 (12.04, 14.77)	0.532
SBP (mmHg)^a^	90.14 ± 16.14	90.18 ± 16.22	90.06 ± 16.02	0.942
DBP (mmHg)	52.00 (44.00, 61.00)	52.00 (45.00, 61.00)	50.00 (41.00, 61.00)	0.366
Heart rate (beats per minute)^a^	161.00 (147.00, 175.00)	160.00 (147.00, 172.00)	164.00 (147.50, 179.00)	0.398
Modified Ross classification (*n*, %)				0.649
Mild (3 ~ 6) scores	239 (58.7)	171 (60.2)	68 (55.3)	
Moderate (7 ~ 9) scores	138 (33.9)	93 (32.7)	45 (36.6)	
Severe (10 ~ 12) scores	30 (7.4)	20 (7.0)	10 (8.1)	
Comorbidities				
Shock (*n*, %)	35 (8.6)	26 (9.2)	9 (7.3)	0.544
Malignant arrhythmia (*n*, %)	19 (4.7)	12 (4.2)	7 (5.7)	0.520
MODS (*n*, %)	5 (1.2)	4 (1.4)	1 (0.8)	1.000
PHC (*n*, %)	7 (1.7)	4 (1.4)	3 (2.4)	0.436
Echocardiographic data				
AO (mm)	11.00 (9.00, 13.00)	11.00 (9.00, 13.00)	11.00 (9.00, 13.00)	0.896
LA (mm)	18.00 (15.00, 21.00)	18.00 (15.00, 21.00)	18.00 (15.00, 21.00)	0.899
LVEDD (mm)	26.00 (22.00, 31.00)	26.00 (21.00, 31.00)	26.00 (24.00, 30.00)	0.521
LVESD (mm)	16.00 (13.00, 19.00)	16.00 (13.00, 19.00)	15.00 (14.00, 19.00)	0.835
IVS (mm)	5.50 (5.00, 7.00)	5.75 (5.00, 6.00)	5.50 (4.75, 7.00)	0.636
LVPW (mm)	5.00 (4.00, 6.00)	5.00 (4.38, 6.00)	5.00 (4.00, 7.00)	0.769
RV (mm)	12.00 (10.00, 14.00)	12.00 (11.00, 14.00)	12.00 (9.00, 14.00)	0.459
RVOT (mm)	15.00 (13.00, 18.00)	15.00 (13.00, 18.00)	15.00 (13.00, 18.00)	0.987
PA (mm)	15.00 (12.00, 17.00)	15.00 (12.00, 17.00)	15.00 (12.00, 18.00)	0.558
LVEF (%)	74.00 (69.00, 77.10)	73.50 (69.00, 78.00)	74.00 (68.50, 77.00)	0.351
LVFS (%)	41.00 (37.00, 44.00)	40.00 (37.00, 44.40)	41.00 (37.00, 44.00)	0.337
Laboratory data				
NT-proBNP (*n*, %)				0.072
≤5743.00 pg./ml	300 (73.7)	202 (71.1)	98 (79.7)	
>5743.00 pg./ml	107 (26.3)	82 (28.9)	25 (20.3)	
CK-MB (U/L)	28.00 (21.00, 36.00)	27.00 (21.00, 35.00)	29.00 (20.00, 36.50)	0.650
cTnI (μg/L)	0.04 (0.01, 0.09)	0.03 (0.01, 0.09)	0.04 (0.01, 0.09)	0.696
ALT (U/L)	24.00 (17.00, 35.00)	24.00 (18.00, 35.00)	23.00 (16.00, 35.00)	0.266
AST (U/L)	42.00 (34.00, 56.00)	43.00 (35.00, 56.00)	41.00 (33.00, 54.50)	0.586
Albumin (g/L)	40.20 (37.20, 42.70)	40.35 (37.27, 42.70)	39.90 (37.20, 42.90)	0.626
BUN (mmol/L)	3.43 (2.40, 4.93)	3.37 (2.33, 4.86)	3.65 (2.58, 5.09)	0.268
K (mmol/L)	4.47 (3.99, 4.93)	4.48 (3.96, 4.91)	4.45 (4.05, 4.96)	0.759
Na (mmol/L)^a^	137.00 (134.50, 139.10)	137.00 (134.50, 139.00)	137.40 (134.85, 139.20)	0.226
Ca (mmol/L)	2.36 (2.26, 2.49)	2.35 (2.25, 2.49)	2.40 (2.28, 2.53)	0.151
PT (s)	12.00 (11.00, 13.30)	12.00 (10.90, 13.20)	12.10 (11.20, 13.35)	0.381
APTT (s)	34.80 (30.65, 39.70)	34.85 (31.27, 39.62)	34.60 (30.45, 41.10)	0.701
WBC (×10^9^/L)	9.76 (7.53, 13.09)	9.96 (7.67, 13.46)	9.40 (7.47, 12.27)	0.092
RBC (×10^12^/L)^a^	3.94 ± 0.76	3.96 ± 0.79	3.90 ± 0.69	0.462
BPC (×10^9^/L)	344.30 (278.25, 428.00)	340.60 (278.43, 420.13)	357.60 (279.80, 429.45)	0.439
Hemoglobin (g/l)	100.60 (92.00, 112.00)	100.50 (91.90, 111.85)	101.00 (93.20, 112.20)	0.560
Hematocrit (%)	0.32 (0.29, 0.35)	0.32 (0.28, 0.35)	0.32 (0.29, 0.35)	0.503
RDW-CV (*100)	17.00 (15.00, 19.00)	16.00 (15.00, 19.00)	17.00 (15.00, 20.00)	0.029
Lactate (mmol/L)	1.91 (1.28, 2.63)	1.91 (1.28, 2.62)	1.94 (1.22, 2.69)	0.892
PCT (ng/ml)	0.14 (0.08, 0.33)	0.15 (0.08, 0.33)	0.12 (0.07, 0.34)	0.484
CRP (*n*, %)				0.339
<10 mg/L	356 (87.5)	247 (87.0)	109 (88.6)	
10 ~ 25 mg/L	19 (4.7)	16 (5.6)	3 (2.4)	
>25 mg/L	32 (7.9)	21 (7.4)	11 (8.9)	
HsCRP (*n*, %)				0.244
<1 mg/L	287 (70.5)	200 (70.4)	87 (70.7)	
1 ~ 3 mg/L	54 (13.3)	42 (14.8)	12 (9.8)	
>3 mg/L	66 (16.2)	42 (14.8)	24 (19.5)	
Therapeutic methods				
Thoracotomy (*n*, %)	135 (33.2)	93 (32.7)	42 (34.1)	0.783
MV (*n*, %)	200 (49.1)	138 (48.6)	62 (50.4)	0.737

BMI, body mass index; SBP/DBP, systolic/diastolic blood pressure; MODS, multiple organ dysfunction syndrome; PHC, pulmonary hypertensive crisis; AO, aod aortic dimension; LA, left atrial diameter; LVEDD, left ventricular end-diastolic dimension; LVESD, left ventricular end-systolic diameter; IVS, interventricular septal thickness; LVPW, left ventricular posterior wall thickness; RV, right ventricular diameter; RVOT, outflow tract of right ventricle; PA, pulmonary artery diameter; LVEF, left ventricular ejection fraction; LVFS, left ventricular fractional shortening; NT-proBNP, N-terminal pro-brain natriuretic peptide; CK-MB, creatine kinase isoenzyme; cTnI, cardiac troponin I; ALT, alanine aminotransferase; AST, aspartate aminotransferase; BUN, blood urea nitrogen; PT, prothrombin time; APTT, activated partial thromboplastin time; WBC/RBC, white/red blood cell; BPC, blood platelet count; RDW-CV, coefficient of variation of red cell distribution width; PCT, procalcitonin; CRP, C-reactive protein; HsCRP, high-sensitivity C-reactive protein; MV, mechanical ventilation.

^a^Data are presented as mean ± standard deviation, while others are reported as median (interquartile range) or *n* (%).

Among the 135 patients who underwent cardiac surgery, 56 (41.4%) presented with simple congenital heart defects, mainly VSDs (*n* = 41; 73.2%); whereas compound congenital heart defects were observed in 79 individuals (59.8%) (Supplementary Table S3). The training and validation set had 56 and 28 patient deaths, respectively. To further clarify mortality distribution across key subgroups, absolute numbers of deaths were summarized as follows: among the 135 patients who underwent thoracotomy, 12 (8.89%) died; in contrast, among the 272 patients who did not receive thoracotomy, 72 (26.47%) died. Regarding HF severity (based on modified Ross classification), 23 (9.62%) deaths occurred in the mild HF subgroup (*n* = 239), 29 (21.01%) in the moderate HF subgroup (*n* = 138), and 22 (73.33%) in the severe HF subgroup (*n* = 30). Additionally, 41 (13.67%) deaths were recorded in patients with NT-proBNP ≤5,743 pg./mL (*n* = 300), while 43 (39.99%) deaths occurred in those with NT-proBNP >5,743 pg./mL (*n* = 107). For comorbidities, a notable association with HF severity and mortality was observed: 62.9% (22/35) of patients with shock were classified as severe HF (modified Ross score 10–12), and univariate Cox regression analysis (see Supplementary Table S5) showed shock was strongly associated with increased mortality risk (HR = 6.213, 95% CI: 3.500–11.028, *p* < 0.001). In contrast, malignant arrhythmia (*n* = 19) had no significant prognostic impact (HR = 1.392, 95% CI: 0.435–4.456, *p* = 0.577).

Among the 272 non-surgical patients, the reasons for not undergoing thoracotomy were mutually exclusive and documented in medical records: (1) Preoperative instability (48.5%, 132/272): Severe uncontrolled HF (modified Ross score ≥11, *n* = 89) or refractory shock (*n* = 43) that precluded anesthesia tolerance, despite maximal anti-HF therapy (diuretics + high-dose ACEI + inotropes); (2) Surgical contraindications (29.4%, 80/272): Malignant neoplasms (*n* = 12), severe central nervous system disease (*n* = 18), irreversible pulmonary hypertension (mean pulmonary arterial pressure >75% systemic pressure, *n* = 31), or spontaneously closed small defects (*n* = 19); (3) Family refusal (17.3%, 47/272): Concerns about anesthesia risk (*n* = 23) or financial constraints (*n* = 18); (4) Spontaneous defect closure (4.8%, 13/272): Confirmed by echocardiography 3–6 months after HF diagnosis (all isolated PDA or small VSD).

### 3.2 Development of the prognostic nomogram

**Figure 1:**
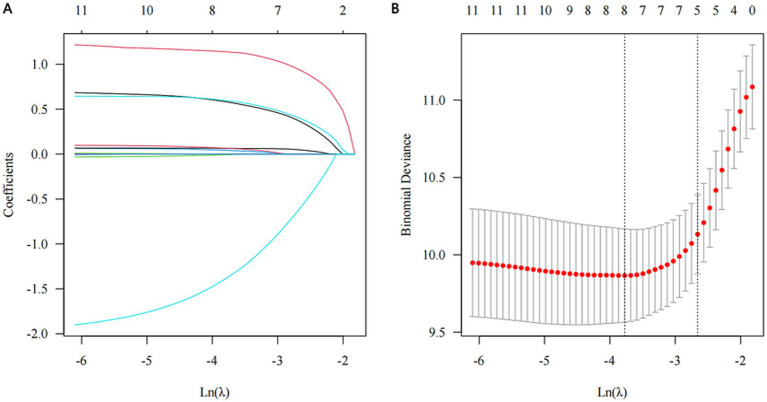
Significant features selection through the least absolute shrinkage and selection operator (LASSO) regression algorithm. **(A)** The optimal parameter (λ) determination through 10-fold cross validation, employing the 1-standard error of the minimum criteria, i.e., *λ* = 0.086, with log(*λ*) = −2.453. **(B)** LASSO coefficient profile, five out of 12 variables with non-zero coefficients chosen by the optimal λ.

**Figure 2:**
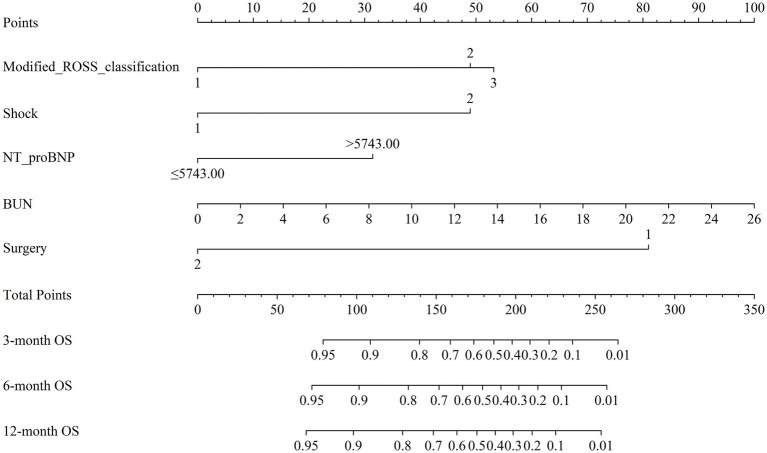
Nomogram for forecasting overall survival (OS) of children with left-to-right (L-R) shunt congenital heart disease (CHD) complicated by heart failure (HF). Note: Different grades or categories of each clinical variable included in the model (Modified ROSS classification, Shock, NT-proBNP, BUN, Surgery) correspond to a certain number of points as shown in the top row. The cumulative points were obtained by summing up the points for each variable, and the total points on the bottom scales were associated with the probabilities of survival at 3, 6, and 12 months. NT-ProBNP, N-terminal pro-brain natriuretic peptide; BUN, blood urea nitrogen.

The results of univariate Cox proportional hazards regression analyses of prognostic factors for overall survival in the training cohort are reported in Supplementary Table S4. Subsequently, we employed the LASSO Cox regression algorithm to screen for significant features (Figure 1; Supplementary Table S5). Finally, independent prognostic variables were determined using multivariate Cox regression (*p* < 0.05). These predictors included the modified Ross classification, elevated levels of NT-proBNP (>5743.00 pg./mL), increased BUN levels, the presence of shock, and undergoing a thoracotomy procedure (Table 2). The findings revealed that the severe HF group exhibited a statistically significant increase in the likelihood of mortality when contrasted with the mild HF group (HR 3.633, *p* = 0.003). When NT-proBNP levels exceeded 5743.00 pg./mL, the mortality risk increased 1.145-fold (HR 2.145, *p* = 0.022). As the concentration of BUN increased, so did the corresponding risk of death (HR 1.098, *p* = 0.019). Furthermore, septic or cardiogenic shock increased the mortality risk in pediatric patients with HF by 2.279-fold (HR 3.279, *p* = 0.001). Notably, among patients in our study who underwent thoracotomy (76.3% of whom received surgery within 2 weeks of HF diagnosis), surgical treatment was associated with a protective effect, reducing mortality risk by 86.0% compared to nonsurgical intervention (HR 0.140, *p* < 0.001). This protective effect may be influenced by factors such as surgical timing and baseline HF severity, and may not be generalizable to patients with extremely severe preoperative conditions. Based on the above outcomes, we have integrated these five predictors. The cumulative scores were obtained by mapping the data points of each variable onto the “Points” axis, resulting in corresponding predictions (21). The nomogram model predicts mortality in children with CHD complicated by HF, providing a valuable tool for assessing OS at 3, 6, and 12 months. Notable advantages of this model include the utilization of the modified Ross classification (with a maximum point of 53), elevated levels of NT-proBNP (>5743.00 pg./mL) (31 points), increased BUN levels (with a maximum point of 100), the presence of shock (49 points), and undergoing a thoracotomy procedure (81 points) (Figure 2). A higher cumulative point is indicative of a poor clinical outcome and an increased likelihood of shorter expected survival.

**Table 2:** Multivariate Cox regression analysis of OS in children with L-R shunt CHD complicated by HF.

Variables	Multivariate analysis		
	HR	95% CI	*P*-value
Modified Ross classification			
Moderate vs. Mild	3.280	1.798–5.986	<0.001
Severe vs. Mild	3.633	1.572–8.397	0.003
Shock (Yes vs. No)	3.279	1.588–6.769	0.001
NT-proBNP (>5,743 vs. ≤5,743; pg./ml)	2.145	1.119–4.111	0.022
BUN (mmol/L)	1.098	1.015–1.187	0.019
Thoracotomy (Yes vs. No)	0.140	0.050–0.394	<0.001

OS, overall survival; L-R, left-to-right; CHD, congenital heart disease; HF, heart failure; HR, hazard ratio; CI, confidence interval.

Absolute numbers of deaths per subgroup for each variable are as follows: (1) Modified Ross classification: Mild HF (3 ~ 6 points): 23 deaths/239 patients; Moderate HF (7 ~ 9 points): 29 deaths/138 patients; Severe HF (10 ~ 12 points): 22 deaths/30 patients. (2) Shock: With shock: 18 deaths/35 patients; Without shock: 66 deaths/372 patients. (3) NT-proBNP: >5,743 pg/mL: 43 deaths/107 patients; ≤5,743 pg/mL: 41 deaths/300 patients. (4) BUN: For every 1 mmol/L increase in BUN, the number of deaths increased progressively (e.g., BUN <3 mmol/L: 15 deaths/148 patients; 3 ~ 5 mmol/L: 38 deaths/176 patients; >5 mmol/L: 21 deaths/83 patients). (5) Thoracotomy: With thoracotomy: 12 deaths/135 patients; Without thoracotomy: 72 deaths/272 patients.

### 3.3 Nomogram validation

**Figure 3:**
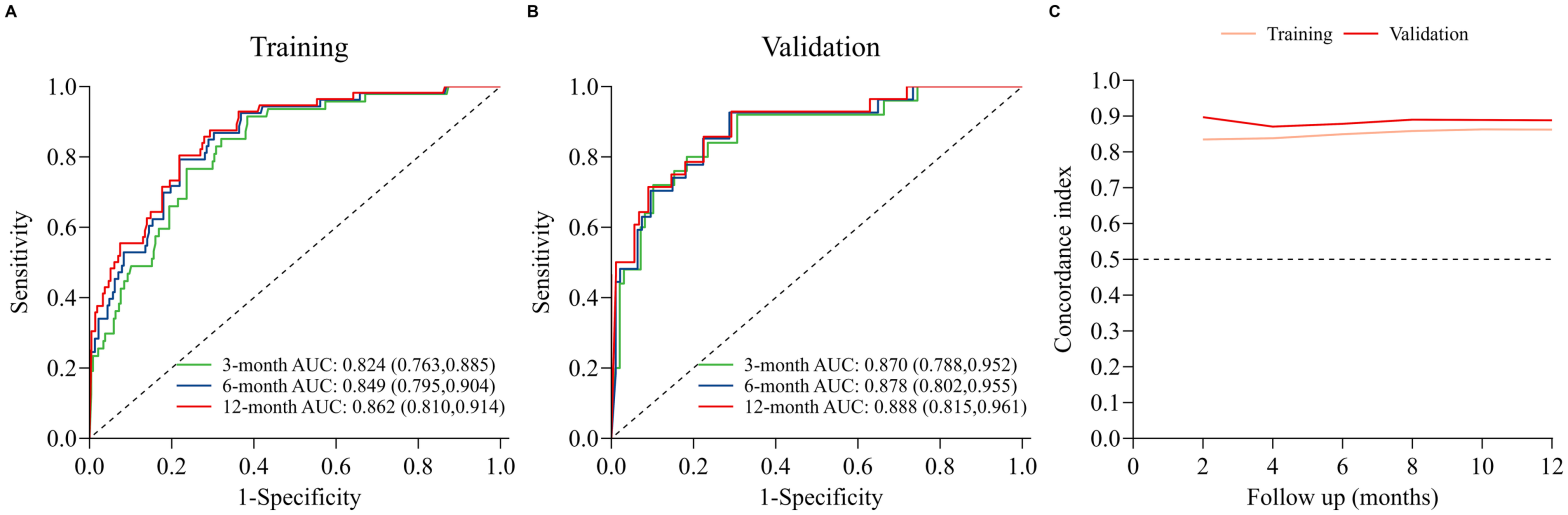
Discriminative performance of the nomogram for OS prediction: **(A)** ROC curves and AUC in training set; **(B)** ROC curves and AUC in validation set; **(C)** time-dependent concordance indexes (C-indexes) in both sets.

**Figure 4:**
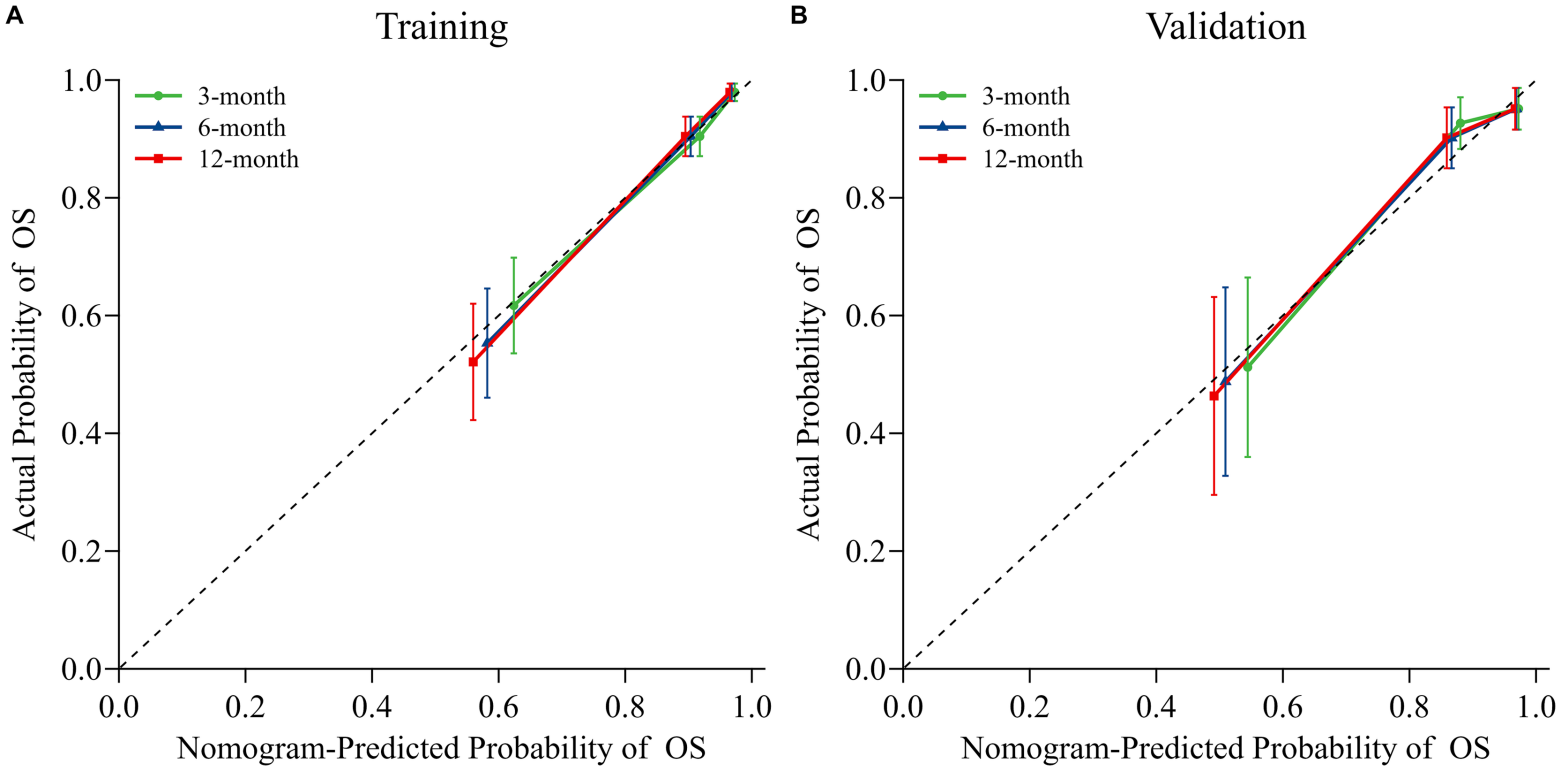
Nomogram calibration curves for predicting OS in both the training **(A)** and validation **(B)** sets. Dotted lines indicate ideal alignment between predicted and observed probabilities, whereas solid blue/orange/red lines depict the observed model of OS at 3-, 6-, and 12-months, respectively.

**Figure 5:**
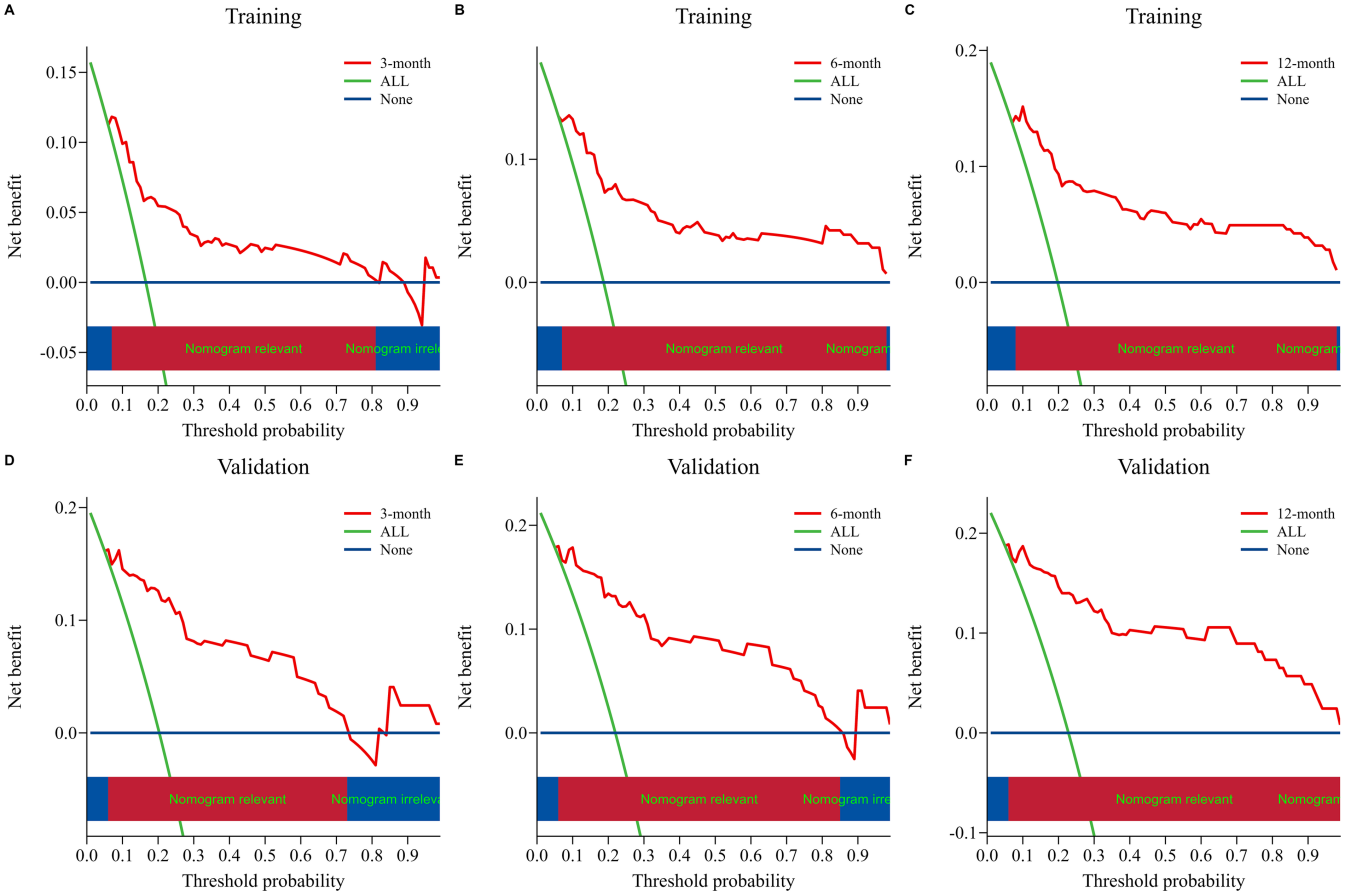
Nomogram decision curve analyses for assessing the clinical utility in predicting OS at 3, 6, and 12 months for both the training set **(A–C)** and validation set **(D–F)**. The net benefit of treating all patients and no patients is represented by the blue and orange lines, respectively. The red lines demonstrate the net benefit of the nomogram at various risk thresholds.

The nomogram model was internally validated using a 1,000 bootstrap resampling method (22). In the validation set, we analyzed clinical data from 123 patients to examine the independent risk factors in the nomogram model. To assess the discriminative ability of a survival prediction model for positive events, the e time-dependent area under the receiver operating characteristic curve and C-index were employed as evaluation metrics (23). The training set yielded area under the curve (AUC) values of 0.824, 0.849, and 0.862 for OS at 3-, 6-, and 12-months, respectively (Figure 3A). Similarly, the corresponding AUCs for the validation set were 0.870, 0.878, and 0.888 (Figure 3B). The model demonstrated good discrimination in accurately predicting survival and mortality outcomes, as evidenced by the AUC values in both sets. Moreover, the C-indexes for both training and validation sets were found to be 0.829 [95% confidence interval (CI) 0.781–0.876] and 0.850 (95% CI 0.789–0.912) respectively, indicating a high level of concordance and a reliable predictive capability (Figure 3C). The calibration curves demonstrated a good fit in predicting the actual 3-, 6-, and 12-month survival rates in both sets, showing a good agreement with the observed survival probabilities (Figure 4). DCA was used to assess the clinical significance associated with the potential outcomes under consideration (24). The DCA curves demonstrated the model’s significant clinical utility in predicting OS across both sets at various time points. In the training set, net clinical benefits were observed for 3-, 6-, and 12-month OS at threshold probabilities ranging from 0.05 to 0.99. Similarly, in the validation set, they were noted for OS when threshold probabilities varied between 0.06 and 0.99 (Figure 5). Consequently, the nomogram model exhibits a commendable predictive capacity and holds significant clinical value.

**Figure 6:**
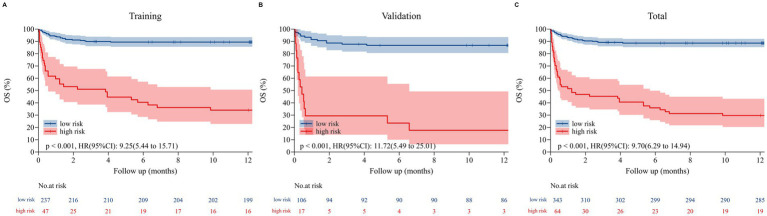
The Kaplan–Meier curves for overall survival (OS) in the training set **(A)**, validation set **(B)**, and total set **(C)**. Those with a total point < 151.82 for OS were classified as low risk, while those with a total point ≥ 151.82 were classified as high risk.

Finally, patients in the training cohort, validation cohort, and Total set were stratified into two risk groups (high risk vs. low risk) using the calculated optimal cutoff for the nomogram scores (152 points for overall survival). Kaplan–Meier curves of Sub-risk stratification based on nomogram model assessment are shown for OS among low-risk and high-risk groups within each set (Figure 6). The optimal cutoff of 153 points for nomogram scores stratified patients into low-risk (total points <153) and high-risk (total points ≥153) subgroups. Absolute numbers of deaths in these subgroups were as follows: in the training set (*n* = 284), 11 deaths occurred in the low-risk subgroup (*n* = 192, 5.73%) and 45 deaths in the high-risk subgroup (*n* = 92, 48.91%); in the validation set (*n* = 123), 5 deaths occurred in the low-risk subgroup (*n* = 85, 5.88%) and 23 deaths in the high-risk subgroup (*n* = 38, 60.53%); in the total cohort (*n* = 407), 16 deaths occurred in the low-risk subgroup (*n* = 277, 5.78%) and 68 deaths in the high-risk subgroup (*n* = 130, 52.31%). The findings indicated that 3-, 6-, and 12-month survival rates were significantly higher for low-risk patients compared to their high-risk counterparts within each set (all *p* < 0.001) (Supplementary Table S6).

### 3.4 Impact of risk stratification and surgical timing on 12-month survival rate

A total of 135 surgical patients were included, with 60 in the high-risk group and 75 in the low-risk group. 12-month survival rates across different risk stratifications and surgical timings are presented in Table 3. In the high-risk group, a significant negative correlation was observed between surgical timing and survival rate (log-rank *χ*^2^ = 18.26, *p* < 0.001). Patients who underwent early surgery (within 1 week) had the highest 12-month survival rate. Those who received intermediate surgery (1–2 weeks) showed a decreased survival rate of 71.4%, while the lowest survival rate was seen in patients with delayed surgery (beyond 2 weeks). In the low-risk group, there was no statistically significant difference in survival rates among different surgical timings (log-rank *χ*^2^ = 2.15, *p* = 0.312).

**Table 3:** Comparison of 12-month survival rates in patients with different risk stratifications and surgical timings.

Risk stratification	Surgical timing	Sample size (n)	Number of deaths (n)	12-Month survival rate (%) [95% CI]	Log-rank Test *P*-value
High-risk group	<1 Week	24	3	87.5 [71.2–95.3]	<0.001
	1–2 Weeks	21	6	71.4 [48.9–85.7]	
	>2 Weeks	15	8	46.7 [22.9–69.4]	
Low-risk group	<1 Week	37	1	97.3 [85.5–99.7]	0.312
	1–2 Weeks	30	1	96.7 [82.8–99.6]	
	>2 Weeks	8	1	87.5 [47.3–98.2]	

### 3.5 Quantification of surgical benefit across different risk groups

Results from the multivariate Cox regression analysis are presented in Table 4. In the high-risk group, the 12-month mortality risk of patients who underwent surgery was significantly lower than that of non-surgical patients. In the low-risk group, surgery still reduced mortality risk, but the magnitude of this benefit was smaller than that in the high-risk group.

**Table 4:** Multivariate cox regression analysis of surgical versus non-surgical patients across different risk stratifications.

Risk stratification	Treatment modality	Sample size (n)	Number of deaths (n)	Adjusted HR [95% CI]	*P*-value
High-risk group	Surgery	60	9	0.13 [0.06–0.29]	<0.001
	No surgery	70	37	1.00 (Reference)	-
Low-risk group	Surgery	75	4	0.36 [0.14–0.92]	0.032
	No surgery	202	12	1.00 (Reference)	-

Adjusted variables include age, modified Ross classification (mild/moderate/severe), and presence of shock.

## 4 Discussion

In this retrospective cohort study, we developed and validated a nomogram model to predict overall survival in pediatric patients with L-R shunt CHD complicated by HF. The model consistently showed superior prognostic performance during 3, 6,12 months of follow-up. By calculating individual nomogram scores, clinicians can categorize children into risk groups with different prognoses and recommend closer clinical care for high-risk pediatric patients.

This study demonstrated that the modified Ross classification, elevated levels of NT-proBNP (>5743.00 pg./mL), increased BUN levels, and the presence of shock were independent risk factors for OS, while thoracotomy was an independent protective prognostic variable significantly associated with improved OS. Besides clinical signs and symptoms, exercise testing, echocardiography, and biomarkers have all been shown to be helpful in stratifying outcomes in children with HF (25, 26). The most frequently utilized scales for classifying HF in pediatric patients include the New York Heart Association (NYHA) classification, the Pediatric Heart Failure Index, and the modified Ross heart failure classification. However, the NYHA classification is not applicable for assessing infants and patients experiencing acute HF. The modified Ross classification can be applied to evaluate the severity of HF in children aged 0–14 years (27). A higher score signifies a greater severity of the disease. Hassan et al. (28) reported that children with HF had significantly higher modified Ross scores than those without HF, suggesting that the utilization of the modified Ross score could potentially serve as a valuable approach for evaluating and predicting severe lower respiratory tract infections in children with concomitant HF. Our study revealed a significantly increased risk of mortality in the severe HF group (with a modified Ross score ranging from 10 to 12).

The NT-proBNP biomarker is crucial for the diagnosis, assessment of severity, evaluation of treatment response, and prediction of prognosis in CHD complicated by HF (25). The concentration of NT-proBNP exhibited a positive correlation with both the severity of HF and the risk of mortality (29–31). Chowdhury et al. (32) observed a statistically significant elevation in the median NT-proBNP value among deceased children with CHD complicated with HF, compared to the survivors (11681.01 pg./mL versus 893.4 pg./mL, *p* < 0.001), suggesting the potential prognostic significance of NT-proBNP levels in hospitalized children with CHD complicated by HF. Our study revealed that over 26.3% of the children exhibited NT-proBNP levels exceeding 5743.00 pg./mL, indicating severe cardiac dysfunction and a 1.14 times increased risk of mortality. The heart and kidneys have a close, interdependent relationship, and sudden impairment of cardiac function can lead to acute kidney injury. Therefore, increased BUN levels are highly sensitive indicators in patients with HF and have predictive value in assessing poor prognosis (33, 34). Furthermore, a machine learning-based study reported that elevated levels of BUN and NT-proBNP were identified as significant prognostic factors for readmission or mortality shortly after admission in adult patients with HF (35). As found in our study, these two indicators showed a correlation with higher mortality rates among children with HF. However, discrepancies in the threshold values of predictors influencing prognosis in patients with HF may arise because of variations in sample size, demographic characteristics of the study population, and etiologies of HF.

During the progression of HF, septic and cardiogenic shock are common complications. When septic shock occurs in children with CHD complicated with HF, inflammatory cytokines contribute to a systemic inflammatory response, which exacerbates the deterioration of cardiac function (36). Cardiogenic shock represents the most severe manifestation of HF, exhibiting impaired cardiac pump function, diminished cardiac output, and the occurrence of tissue ischemia and hypoxia, leading to disturbances in microcirculation (37). Treatment at this stage becomes increasingly challenging due to an extremely high mortality rate (with in-hospital mortality rates for adults reaching up to 27–51%). Based on our study, children with L-R shunt CHD complicated with HF who experienced septic or cardiogenic shock had a 2.279-fold increased risk of mortality compared to children without these complications, emphasizing the importance of promptly identifying symptoms and intervening in a timely manner to mitigate the risk of mortality in such patients.

Although there have been remarkable breakthroughs in the clinical research of CHD, surgical treatment remains necessary for large defects and complex congenital heart defects (38). Continued advancements in preoperative diagnostic approaches, anesthetic techniques, surgical expertise, and extracorporeal circulation technology, along with improved postoperative care and interdisciplinary teamwork, have progressively reduced the mortality associated with CHD surgery (39). As a result, age and weight restrictions for patients requiring surgery have been relaxed. Research findings have indicated that younger pediatric patients with CHD have higher mortality rates, and the postoperative mortality rate is highest in complex mixed congenital heart defects and lowest in L-R shunts (40, 41). Surgery has significantly improved the prognosis of patients with CHD, including those with complex CHD (42). Our study also showed that surgical treatment acts as a protective factor for the prognosis of L-R shunt CHD complicating HF, substantially reducing the risk of death. This finding aligns with the broader landscape of pediatric HF management described by Olsen and Miyamoto (43).

A key strength of this study is the use of a nomogram as the prognostic assessment tool. This tool addresses critical limitations of existing predictive methods when evaluating pediatric patients with left-to-right shunt CHD complicated by HF. Moreover, the robust performance of our model—with a C-index of 0.829 in the training set and 0.850 in the validation set—further confirms the rationality of this choice.

First, compared with traditional risk scoring systems (such as unweighted summation of risk factors), the nomogram constructed in this study quantifies the differential contribution of each prognostic factor based on Cox regression and LASSO selection. For example, thoracotomy (weighted 81 points) and BUN (maximum weight 100 points) have higher weights than NT-proBNP > 5,743 pg./mL (31 points). This weight difference is consistent with their HR: HR for thoracotomy is 0.140, and HR for BUN is 1.098. This weighted design avoids the oversimplification of equal risk weighting, which is crucial for accurate risk stratification of heterogeneous pediatric CHD populations. In contrast, traditional scoring systems may merge these factors into a single count item, leading to misclassification of high-risk and low-risk cases. However, the OS of the high-risk and low-risk groups defined by the nomogram in this study shows significant differences, with all *p*-values < 0.001.

Second, the performance of this nomogram is superior to single-biomarker or single-clinical-index models (such as using only NT-proBNP to predict survival). Our model integrates five independent factors: modified Ross classification, NT-proBNP, BUN, shock, and thoracotomy. These factors collectively cover the multifactorial pathophysiological mechanisms of HF prognosis in this population: cardiac function severity, neuroendocrine activation, cardiorenal interaction, hemodynamic instability, and therapeutic intervention. This comprehensiveness avoids the bias of single-index models. For instance, in a child with normal NT-proBNP but severe shock (HR = 3.279), a model based solely on NT-proBNP would underestimate the risk, while the nomogram in this study can correctly classify the child as high-risk. This limitation of single-biomarker models aligns with the consensus from pediatric HF biomarker research. Senekovič Kojc and Marčun Varda (44). Additionally, the high AUC values of the model at 3, 6, and 12 months—ranging from 0.824 to 0.888—further confirm that multi-variable integration improves predictive accuracy.

Third, unlike machine learning black box models, the nomogram in this study has complete transparency. Clinicians can trace the contribution of each patient’s data to the total risk score. For example, a 4-month-old infant with a modified Ross score of 10, a BUN level of 6 mmol/L, and no surgery has a total risk score of approximately 180 points, corresponding to a 12-month survival rate of < 50%. Clinicians can also explain the basis of risk stratification to the children’s families. This interpretability is crucial in pediatric clinical practice.

Finally, the good calibration and clinical utility of the nomogram further prove its superiority over other tools. Many machine learning models perform well in the training set but are difficult to generalize to external data. In contrast, the nomogram in this study maintains consistent performance in both the training and validation sets. Its net clinical benefit shows that the model’s value goes beyond the two extreme strategies of “treating all patients” or “treating no patients.” This practical advantage is of great significance for the allocation of medical resources.

To further confirm that potential confounding variables do not substantially interfere with the model’s core conclusions, we conducted additional stratified analyses based on two factors that were excluded from the final model but may correlate with prognosis: “presence of concurrent pneumonia” and “WAZ < −2 (severe malnutrition).” After stratification, the direction and magnitude of hazard ratios (HR) for the five predictive factors in the original model (modified Ross classification, NT-proBNP, BUN, shock, and thoracotomy) remained stable without significant changes. For example, in the stratum with concurrent pneumonia, the HR for severe modified Ross classification was 3.582 (95% CI: 1.491–8.597, *p* = 0.004); in the stratum without pneumonia, the HR was 3.671 (95% CI: 1.532–8.798, *p* = 0.003). Similar consistency was observed in the stratification by “WAZ < −2”: the HRs of all five predictors in both the severe malnutrition and non-severe malnutrition subgroups were consistent with the overall model results (all P for interaction > 0.05). These findings indicate that even after accounting for potential confounding effects of concurrent pneumonia and severe malnutrition, the associations between the core predictive factors and overall survival remain reliable, further supporting the robustness of the nomogram model.

The higher mortality in our study (8.89% surgical, 26.47% non-surgical) compared to the STAT score (2.3–4.5% for low-to-moderate risk) stems from three key differences: (1) Target population: The STAT score includes asymptomatic CHD patients, while our study only includes those with severe HF (modified Ross score ≥3), which increases mortality by 3–5-fold; (2) Outcome timeframe: The STAT score measures 30-day postoperative mortality, while our study reports 12-month OS (42% of non-surgical deaths occur 3–12 months post-diagnosis); (3) Patient severity: Our cohort has a younger median age (4.33 vs. 6 months in STAT) and higher proportion of complex defects (49.8% vs. <30% in STAT low-risk). Notably, for surgical patients matching STAT Class 3 (*n* = 52), our 30-day mortality was 5.8%, consistent with the STAT score’s 4.5–6.2%.

However, our study also has several limitations that require careful consideration when interpreting the findings. Firstly, as a retrospective investigation relying on historical medical records from a single center, inherent selection and information biases cannot be fully eliminated. For selection bias, the study only included patients who met the strict inclusion/exclusion criteria and had complete clinical data, which excluded those with incomplete records or those who sought medical care at other institutions during the study period. This may lead to a sample that is not fully representative of the broader population of pediatric patients with L-R shunt CHD and HF. For instance, patients with extremely mild symptoms who did not require hospitalization, or those with severe comorbidities who were transferred to intensive care units outside our hospital, were not captured. For information bias, the accuracy of data (such as the timing of HF diagnosis or the severity of shock) depended on the completeness and objectivity of retrospective documentation; subtle variations in how clinicians recorded these details may have introduced unintended errors into the analysis. Additionally, unmeasured confounding factors—including family socioeconomic status, long-term nutritional status, and a history of minor infections —were not accounted for due to the constraints of retrospective data collection, which may have subtly influenced the association between the identified risk factors and prognosis.

Secondly, certain variables with potential prognostic value were excluded from the analysis due to a missing data rate surpassing 20%, which may have limited the comprehensiveness of the model. Specifically, fasting blood glucose and high-sensitivity troponin (hs-cTnI) were excluded: fasting blood glucose, which reflects metabolic status and may correlate with cardiac energy metabolism in HF, had 28.3% of data missing (115/407 cases) primarily because fasting samples were not routinely collected for critically ill infants; hs-cTnI, a more sensitive marker of myocardial injury than conventional cTnI, had 24.6% missing data (100/407 cases) due to variations in laboratory testing protocols during the 12-year study period. Furthermore, novel molecular markers with emerging prognostic relevance in pediatric HF—such as Neuropeptide Y and soluble suppression of tumorigenicity 2 —were not considered. But their absence in our historical records prevented their inclusion, potentially underestimating the model’s predictive accuracy.

Another specific limitation pertains to the exclusion criterion for VSDs. A specific limitation of the VSD exclusion criterion in this study lies in the use of two-dimensional diameter (<5 mm) rather than restrictive hemodynamic characteristics, such as peak flow velocity across the defect (Vmax >4 m/s). For elongated VSDs, transesophageal echocardiography may underestimate the actual defect size when relying solely on 2D measurements—this is because the elongated morphology of such defects can appear smaller in standard imaging planes, leading to potential misclassification of clinically significant lesions. Notably, Vmax is a more direct indicator of hemodynamic relevance compared to 2D diameter: it quantifies the flow velocity through the defect, directly reflecting the degree of shunt restriction and the associated risk of cardiac overload, whereas 2D diameter only provides a static anatomical assessment that does not account for functional impact. However, due to inherent limitations of retrospective data collection, Vmax was not routinely documented in the early phase of the study (August 2012 to December 2017). This lack of standardized Vmax data across the entire study period prevented us from integrating this more precise parameter into our VSD classification and exclusion criteria. While our post-hoc review of available echocardiographic reports (for cases with documented Vmax) confirmed a strong correlation between VSD < 5 mm and restrictive shunts (Vmax >4 m/s), the absence of systematic Vmax recording remains a constraint. Future prospective studies focusing on pediatric left-to-right shunt congenital heart disease should prioritize routine measurement and documentation of Vmax to refine VSD classification, thereby enhancing the accuracy of risk stratification and prognostic modeling.

A further limitation is the exclusion of specific CHD subtypes from the model. Clinically, complex left-to-right shunt subtypes (e.g., truncus arteriosus, atrioventricular septal defect) have poorer prognoses than isolated ASD or VSD. Our post-hoc analysis showed that the 12-month mortality rate of complex subtypes was 22.9% (8/35), which was significantly higher than that of isolated ASD (5.2%, 4/77); however, complex subtypes accounted for only 8.6% (35/407) of the total cohort, with truncus arteriosus representing just 2.2% (9/407) of cases. The small sample size resulted in insufficient statistical power to quantify their prognostic weight—after adjusting for HF-related factors (e.g., modified Ross classification, NT-proBNP), the prognostic impact of CHD subtypes became non-significant (VSD vs. ASD: HR = 1.08, 95% CI: 0.62–1.89, *p* = 0.78).

To address this limitation, future studies should prioritize three key directions: (1) expanding the sample size of complex left-to-right shunt subtypes through multicenter collaboration to improve statistical power for quantifying their prognostic value; (2) developing subtype-stratified models to verify whether subtype-specific risk weights are necessary; (3) validating the model in populations with a higher proportion of complex CHD to assess whether incorporating subtypes enhances predictive performance (e.g., C-index or calibration). Importantly, the current model remains clinically valuable: its core predictors (modified Ross classification, NT-proBNP, elevated BUN, shock, and thoracotomy) consistently reflect mortality risk across all left-to-right shunt subtypes, meeting the basic needs of clinical risk stratification.

Furthermore, the limited sample size (*n* = 407) and the absence of external validation further restrict the generalizability and applicability of the model. More importantly, the model was only validated using an internal cohort from the same institution, rather than external cohorts from different regions or hospitals. Given that clinical practices and patient demographics vary across institutions, the model’s performance may decline when applied to external populations.

Consequently, caution must be exercised when interpreting the model’s predictive value in clinical practice. It should be initially applied as an auxiliary tool for risk stratification in populations with characteristics similar to those in our study rather than being generalized to all settings. To improve the detection of high-risk children with L-R shunt CHD complicated by HF, forthcoming optimization endeavors should prioritize: (1) expanding the sample size through multicenter collaboration to enhance statistical power and reduce selection bias; (2) refining variable selection criteria by prospectively collecting data on previously excluded variables (e.g., fasting blood glucose, hs-cTnI) and novel molecular markers; and (3) conducting rigorous external validation across diverse clinical settings (e.g., urban vs. rural hospitals, pediatric vs. general hospitals) to confirm the model’s robustness. These steps will facilitate more timely and targeted clinical interventions, potentially reducing mortality rates in this high-risk population.

A notable contextual constraint on the generalizability of our findings stems from the high homogeneity of anti-heart failure (HF) pharmacotherapeutic regimens in our cohort. As detailed in Section 2.3, 92.1% (375/407) of enrolled patients received standardized anti-HF treatment, defined as a combination of diuretics plus angiotensin-converting enzyme inhibitors (ACEI) or angiotensin II receptor blockers (ARB), with a subset additionally receiving *β*-blockers. This standardized approach was a consequence of our institutional clinical practice guidelines for pediatric L-R shunt CHD with HF during the study period (2012–2024), which minimized heterogeneity in pharmacotherapy. Univariate analysis confirmed that variations in drug combinations (e.g., ACEI vs. ARB, addition of β-blockers) had no significant impact on overall survival (OS; *p* = 0.615), leading to the exclusion of “pharmacotherapeutic regimens” from the final nomogram model.

This treatment homogeneity, while reducing confounding by drug type in our model, also limits the applicability of our findings to populations with different therapeutic backgrounds. Specifically, our nomogram and risk stratification (e.g., high-risk: nomogram score ≥153; low-risk: score <153) were developed and validated in patients receiving the aforementioned standardized regimen. They may not accurately predict prognosis for pediatric patients with L-R shunt CHD and HF who receive non-standard treatments, such as: (1) Palliative care only (e.g., symptom-directed fluid management without ACEI/ARB, due to severe renal dysfunction or hypotension); (2) Novel anti-HF agents (e.g., sodium-glucose cotransporter 2 inhibitors, which have recently been explored in pediatric HF but were not available or routinely used in our cohort); or (3) Discontinued or suboptimal standardized therapy (e.g., non-adherence to ACEI due to family refusal or adverse effects).

The absence of pharmacotherapeutic variability in our sample also means our model cannot quantify how deviations from standardized treatment might modify the prognostic value of our key predictors. For example, it remains unclear whether the protective effect of thoracotomy (HR = 0.140, 95% CI: 0.050–0.394) or the risk associated with elevated BUN (HR = 1.098 per 1 mmol/L increase) would persist in patients not receiving ACEI/ARB. This limitation underscores the need for caution when applying our nomogram in clinical settings where standardized anti-HF therapy is not the standard of care.

Future studies aiming to enhance the model’s generalizability should prioritize enrolling patients with diverse pharmacotherapeutic profiles, including those receiving non-standard or emerging anti-HF treatments. This would allow for the integration of “treatment regimen” as a potential covariate in prognostic modeling, and enable subgroup analyses to validate whether the nomogram’s performance is consistent across different therapeutic strategies. Until such data are available, clinicians should interpret our model’s predictions within the context of a patient’s adherence to and eligibility for standardized anti-HF therapy, and consider adjusting risk assessments for those with non-standard treatment trajectories.

## 5 Conclusion

In this prognostic cohort study, the modified Ross classification, NT-proBNP, BUN, shock and the thoracotomy procedure, were integrated into the nomogram model for predicting overall survival for children with L-R shunt CHD complicated with HF. This model demonstrated good discrimination and calibration capabilities and has great potential for application in guiding clinical monitoring and improving long-term survival outcomes in patients.

## Data Availability

The original contributions presented in the study are included in the article/Supplementary material, further inquiries can be directed to the corresponding author.
