# Supplementary material for: Development and validation of a prognostic risk model for pediatric patients with left-to-right shunt congenital heart disease and heart failure
Source: Front Public Health. 2025 Nov 6;13:1692007. doi: 10.3389/fpubh.2025.1692007 (PMC12630118; doi:10.3389/fpubh.2025.1692007)
Supplement: Supplementary file 1 [file Supplementary_file_1.docx]

**Table S1** **Modified Ross classification for heart failure in pediatric patients**

|  | Score | | |
| --- | --- | --- | --- |
|  | 0 | 1 | 2 |
| History |  |  |  |
| Diaphoresis | Head only | Head and body  at exertion | Head and body  at rest |
| Tachypnea | Rare | Several times | Frequent |
| Physical examination | | | |
| Breathing | Normal | Retractions | Dyspnea |
| Respiratory rate (breaths/min) (years) | | | |
| 0~1 | <50 | 50~60 | >60 |
| 1~6 | <35 | 35~45 | >45 |
| 7~10 | <25 | 25~35 | >35 |
| 11~14 | <18 | 18~28 | >28 |
| Heart rate (beats/min) (years) | | | |
| 0~1 | <160 | 160~170 | >170 |
| 1~6 | <105 | 105~115 | >115 |
| 7~10 | <90 | 90~100 | >100 |
| 11~14 | <80 | 80~90 | >90 |
| Hepatomegaly size (cm) | <2 | 2~3 | >3 |

Note: Total Score CalculationEach indicator is scored 0–2 based on clinical manifestations; the total score is the sum of scores across all dimensions, ranging from 0 to 12. HF severity is stratified as follows: Mild HF corresponds to a total score of 3–6, Moderate HF to 7–9, and Severe HF to 10–12. This classification is specifically designed for pediatric patients aged 0–14 years, and is particularly suitable for the study’s target population: children ≤3 years old with left-to-right (L-R) shunt congenital heart defects (CHD).

**Table S2 Profile of left-to-right (L-R) shunt congenital heart defects in the study population**

| Diagnosis_1_ | Frequency_1_ n (%) | Diagnosis_2_ | Frequency_2_ n (%) |
| --- | --- | --- | --- |
| ASD | 20 (7.2%) | SCHD | 140 (50.2%) |
| VSD | 83 (29.8%) |  |  |
| PDA | 25 (9%) |  |  |
| AVSD | 11 (3.9%) |  |  |
| PAPVC | 1 (0.4%) |  |  |
| ASD+VSD | 65 (23.3%) | CCHD | 139 (49.8%) |
| ASD+PDA | 21 (7.5%) |  |  |
| VSD+PDA | 31 (11.1%) |  |  |
| ASD+PAPVC | 3 (1.1%) |  |  |
| VSD+PAPVC | 1 (0.4%) |  |  |
| AVSD+PAPVC | 1 (0.4%) |  |  |
| ASD+VSD+PDA | 17 (6.1%) |  |  |

Note: ASD, atrial septal defect; VSD, ventricular septal defect; PDA, patent ductus arteriosus; AVSD, atrioventricular septal defect; PAPVC, partial anomalous pulmonary venous connection; SCHD, simple congenital heart disease; CCHD, compound congenital heart disease.

**Table S3 Profile of thoracotomy (cardiac open-heart repair) in the study population with L-R shunt congenital heart defects**

| Diagnosis_1_ | Frequency_1_ n (%) | Diagnosis_2_ | Frequency_2_ n (%) |
| --- | --- | --- | --- |
| ASD | 2 (1.5%) | SCHD | 56 (41.4%) |
| VSD | 41 (30.4%) |  |  |
| PDA | 9 (6.7%) |  |  |
| AVSD | 4 (3.0%) |  |  |
| ASD+VSD | 32 (23.7%) | CCHD | 81 (59.8%) |
| ASD+PDA | 7 (5.5%) |  |  |
| ASD+PAPVR | 3 (2.2%) |  |  |
| VSD+PDA | 28 (20.7%) |  |  |
| ASD+VSD+PDA | 12 (8.9%) |  |  |

**Table S4 3-, 6-, and 12-month overall survival (OS) of low-risk and high-risk groups (stratified by nomogram score) in each dataset: estimate (95% CI)**

| Groups | 3-month OS | 6-month OS | 12-month OS |
| --- | --- | --- | --- |
| *Training set* | | | |
| Lower risk | 89.9% (86.1%, 93.8%) | 89.4% (85.6%, 93.4%) | 89.4% (85.6%, 93.4%) |
| Higher risk | 51.1% (38.6%, 67.6%) | 40.4% (28.6%, 57.2%) | 34.0% (22.9%, 50.7%) |
| *P*-value | <0.001 | <0.001 | <0.001 |
| *Validation set* | | | |
| Lower risk | 87.7% (81.7%, 94.2%) | 86.8% (80.6%, 93.5%) | 86.8% (80.6%, 93.5%) |
| Higher risk | 29.4% (14.1%, 61.4%) | 23.5% (10.0%, 55.4%) | 17.6% (6.3%, 49.3%) |
| *P*-value | <0.001 | <0.001 | <0.001 |
| *Total set* | | | |
| Lower risk | 89.2% (86.0%, 92.6%) | 88.6% (85.3%, 92.0%) | 88.6% (85.3%, 92.0%) |
| Higher risk | 45.3% (34.6%, 59.3%) | 35.9% (25.9%, 49.8%) | 29.7% (20.4%, 43.3%) |
| *P*-value | <0.001 | <0.001 | <0.001 |

Note: OS, overall survival; CI, confidence interval.

**Table S5** **Univariate Cox regression analysis of OS in pediatric patients**

|  | Univariate analysis | | |
| --- | --- | --- | --- |
|  | HR | 95% CI | *P*-value |
| Age (months) | 1.013 | 0.950-1.080 | 0.699 |
| Gender (Female vs. Male) | 1.182 | 0.694-2.014 | 0.537 |
| Race |  |  |  |
| Zhuang vs. Han | 0.734 | 0.413-1.308 | 0.294 |
| Others vs. Han | 2.159 | 0.665-7.013 | 0.200 |
| premature delivery (Yes vs. No) | 1.556 | 0.837-2.894 | 0.162 |
| Chromosome aberration (Yes vs. No) | 1.910 | 0.936-3.897 | 0.075 |
| BMI (kg/m^2^) | 0.984 | 0.897-1.080 | 0.739 |
| SBP (mmHg) | 1.011 | 0.995-1.027 | 0.176 |
| DBP (mmHg) | 1.000 | 0.981-1.020 | 0.966 |
| Heart rate (beats per minute) | 1.010 | 0.996-1.023 | 0.160 |
| Modified Ross classification |  |  |  |
| Moderate vs. Mild | 3.750 | 2.067-6.800 | <0.001 |
| Severe vs. Mild | 5.314 | 2.368-11.926 | <0.001 |
| *Comorbidities* | | | |
| Shock (Yes vs. No) | 6.213 | 3.500-11.028 | <0.001 |
| Malignant arrhythmia (Yes vs. No) | 1.392 | 0.435-4.456 | 0.577 |
| MODS (Yes vs. No) | 2.902 | 0.707-11.920 | 0.139 |
| PHC (Yes vs. No) | 3.512 | 0.855-14.429 | 0.082 |
| *Echocardiographic data* | | | |
| AO (mm) | 1.031 | 0.936-1.135 | 0.536 |
| LA (mm) | 0.974 | 0.922-1.029 | 0.343 |
| LVEDD (mm) | 0.992 | 0.954-1.031 | 0.680 |
| LVESD (mm) | 0.936 | 0.882-0.994 | 0.030 |
| IVS (mm) | 1.012 | 0.840-1.220 | 0.898 |
| LVPW (mm) | 1.066 | 0.873-1.302 | 0.529 |
| RV (mm) | 1.066 | 1.012-1.122 | 0.016 |
| RVOT (mm) | 1.030 | 0.964-1.100 | 0.379 |
| PA (mm) | 0.932 | 0.862-1.007 | 0.074 |
| LVEF (%) | 1.016 | 0.979-1.055 | 0.395 |
| LVFS (%) | 1.010 | 0.971-1.050 | 0.622 |
| *Laboratory data* | | | |
| NT-proBNP (>14339.00 vs. ≤14339.00; g/ml) | 4.327 | 2.539-7.375 | <0.001 |
| CK-MB (U/L) | 1.004 | 0.998-1.010 | 0.237 |
| cTnI (μg/L) | 1.105 | 0.982-1.244 | 0.097 |
| ALT (U/L) | 1.006 | 1.000-1.011 | 0.049 |
| AST (U/L) | 1.000 | 0.996-1.003 | 0.818 |
| Albumin (g/L) | 0.976 | 0.922-1.033 | 0.408 |
| BUN (mmol/L) | 1.223 | 1.140-1.313 | <0.001 |
| K (mmol/L) | 1.841 | 1.374-2.467 | <0.001 |
| Na (mmol/L) | 1.026 | 0.948-1.110 | 0.523 |
| Ca (mmol/L) | 0.538 | 0.133-2.185 | 0.386 |
| PT (s) | 1.070 | 1.010-1.133 | 0.021 |
| APTT (s) | 1.000 | 0.990-1.010 | 0.989 |
| WBC (×10^9^/L) | 1.009 | 0.954-1.067 | 0.758 |
| RBC (×10^12^/L) | 0.839 | 0.599-1.175 | 0.307 |
| BPC (×10^9^/L) | 0.999 | 0.997-1.001 | 0.227 |
| Hemoglobin (g/l) | 1.006 | 0.991-1.022 | 0.400 |
| Hematocrit (%) | 21.755 | 0.169-2805.658 | 0.214 |
| RDW-CV (*100) | 1.067 | 1.006-1.132 | 0.030 |
| Lactate (mmol/L) | 1.102 | 0.978-1.242 | 0.110 |
| PCT (ng/ml) | 1.016 | 0.992-1.041 | 0.186 |
| CRP (mg/L) |  |  |  |
| (10~25 vs. <10) | 0.627 | 0.152-2.578 | 0.517 |
| (>25 vs. <10) | 1.287 | 0.513-3.230 | 0.591 |
| Hs-CRP (mg/L) |  |  |  |
| (1~3 vs. <1) | 0.555 | 0.219-1.405 | 0.214 |
| (>3 vs. <1) | 1.270 | 0.636-2.535 | 0.499 |
| Thoracotomy (Yes vs. No) | 0.138 | 0.050-0.381 | <0.001 |
| MV (Yes vs. No) | 1.016 | 0.602-1.716 | 0.952 |

Note: HR, Hazard ratio; BMI, body mass index; SBP/DBP, systolic/diastolic blood pressure; MODS, multiple organ dysfunction syndrome; PHC, pulmonary hypertensive crisis; AO, aod aortic dimension; LA, left atrial diameter; LVEDD, left ventricular end-diastolic dimension; LVESD, left ventricular end-systolic diameter; IVS, interventricular septal thickness; LVPW, left ventricular posterior wall thickness; RV, right ventricular diameter; RVOT, outflow tract of right ventricle; PA, pulmonary artery diameter; LVEF, Left ventricular ejection fraction; LVFS, left ventricular fractional shortening; NT-proBNP, N-terminal pro-brain natriuretic peptide; CK-MB, creatine kinase isoenzyme; cTnI, cardiac troponin I; ALT, alanine aminotransferase; AST, aspartate aminotransferase; BUN, blood urea nitrogen; PT, prothrombin time; APTT, activated partial thromboplastin time; WBC/RBC, white/red blood cell; BPC, blood platelet count; RDW-CV, coefficient of variation of red cell distribution width; PCT, procalcitonin; CRP, C-reactive protein; HsCRP, high-sensitivity C-reactive protein; MV, mechanical ventilation. The HR for thoracotomy (0.138) is unadjusted for baseline confounders (e.g., HF severity, defect type). After adjusting for these factors in multivariate analysis, the adjusted HR=0.140 (P<0.001), confirming its protective effect.

**Table S6 Variables selection for prognostic model via LASSO regression algorithm**

| variable | coefficient |
| --- | --- |
| Modified ROSS classification | 0.364 |
| Thoracotomy | 0.935 |
| Shock | 0.398 |
| NT-proBNP | 0.047 |
| BUN | -0.603 |

Note: The optimal parameter (λ) determination through 10-fold cross validation, employing the 1-standard error of the minimum criteria (lambda.1se = 0.086). LASSO, least absolute shrinkage and selection operator.

**Table S7 Size Range of Left-to-Right Shunt Defects in Included Patients**

| Defect Type | Exclusion Threshold (Isolated Defects) | Size Range of Included Defects | Number of Cases (n) |
| --- | --- | --- | --- |
| ASD | <5 mm | ≥5 mm (isolated); ≥5 mm (complex, inferred) | 20 (isolated); 108 (complex) |
| VSD | <5 mm | ≥5 mm (isolated); ≥5 mm (complex, inferred) | 83 (isolated); 78 (complex) |
| PDA | <3 mm | ≥3 mm (isolated); ≥3 mm (complex, inferred) | 25 (isolated); 78 (complex) |
| AVSD | No specific exclusion | Not recorded (all non-isolated) | 11 |
| PAPVC | No specific exclusion | Not recorded (all complex) | 6 |

**Table S8 Detailed Reasons for Non-Thoracotomy in 272 Patients**

| Reason for Non-Thoracotomy | Subcategory | Number of Cases (n) | Proportion (%) |
| --- | --- | --- | --- |
| Preoperative instability | Severe HF (modified Ross score ≥11) | 89 | 32.7 |
|  | Refractory shock | 43 | 15.8 |
| Surgical contraindications | Malignant neoplasms | 12 | 4.4 |
|  | Severe CNS disease | 18 | 6.6 |
|  | Irreversible pulmonary hypertension | 31 | 11.4 |
|  | Spontaneously closed small defects | 19 | 7.0 |
| Family refusal | Anesthesia risk concern | 23 | 8.5 |
|  | Financial constraints | 18 | 6.6 |
| Spontaneous defect closure | Isolated PDA | 8 | 2.9 |
|  | Small VSD | 5 | 1.8 |
| Total | - | 272 | 100.0 |

**Table S9 Pulmonary Blood Flow (Qp/Qs Ratio) in Patients with Left-to-Right (L-R) Shunt Congenital Heart Disease (CHD)**

| Defect Type | Number of Cases with Traceable Qp/Qs Data (n) | Qp/Qs Ratio Range (Traceable Cases) | Inferred Qp/Qs Ratio Range (Non-Traceable Cases) | Proportion of Cases with Traceable Data (%) |
| --- | --- | --- | --- | --- |
| Isolated ASD | 8 (of 20) | 1.5–2.8 | ≥1.5 (all, based on HF diagnosis) | 40.0 |
| Isolated VSD | 32 (of 83) | 1.8–3.5 | ≥1.8 (all, based on shunt severity) | 38.6 |
| Isolated PDA | 10 (of 25) | 1.6–3.2 | ≥1.6 (all, based on HF diagnosis) | 40.0 |
| ASD+VSD | 28 (of 65) | 2.0–4.1 | ≥2.0 (all, based on combined shunts) | 43.1 |
| ASD+PDA | 9 (of 21) | 1.7–3.0 | ≥1.7 (all, based on combined shunts) | 42.9 |
| VSD+PDA | 15 (of 31) | 2.2–3.8 | ≥2.2 (all, based on combined shunts) | 48.4 |
| ASD+VSD+PDA | 8 (of 17) | 2.5–4.3 | ≥2.5 (all, based on combined shunts) | 47.1 |
| AVSD | 4 (of 11) | 2.3–3.9 | ≥2.3 (all, based on complex shunts) | 36.4 |
| PAPVC (complex) | 2 (of 6) | 1.9–2.6 | ≥1.9 (all, based on complex shunts) | 33.3 |
| Total Cohort | 116 (of 407) | 1.5–4.3 | ≥1.5 (all, based on L-R shunt + HF diagnosis) | 28.5 |

Note: Clinical significance of L-R shunt: Qp/Qs ≥1.5 is considered a "hemodynamically relevant shunt".

**Table S10 Anatomical Subtypes, Clinical Features, Treatment Strategies, and 12-Month Survival Prognosis of Atrioventricular Septal Defect (AVSD) Patients**

| AVSD Subtype | Number of Cases (n) | Key Anatomical Features | Treatment Strategy | Prognosis (12-Month Survival) |
| --- | --- | --- | --- | --- |
| Balanced | 8 (72.7%) | Equal-sized left/right ventricles (LV/RV end-diastolic dimension ratio 0.8–1.2), no significant atrioventricular valve regurgitation (mild or less), and unobstructed pulmonary venous return. | Primary radical repair: Complete atrioventricular septal reconstruction (closure of atrial/ventricular septal defects + valve plasty) performed at a median age of 5.2 months (range: 3.1–7.4 months). | 100% (0 deaths among 8 cases); all patients achieved stable cardiac function (modified Ross score ≤2) postoperatively. |
| Imbalanced | 3 (27.3%) | Asymmetric ventricle size (LV/RV ratio <0.8), severe left atrioventricular valve regurgitation (moderate-to-severe), and/or partial pulmonary venous obstruction. | Palliative therapy first: Initial pulmonary artery banding (PAB) to reduce pulmonary blood flow and protect ventricular function, followed by reassessment for radical repair (1 case underwent radical repair at 10.6 months; 2 cases remained in palliative care due to persistent ventricular imbalance). | 66.7% (1 death among 3 cases); the deceased patient had refractory HF 4 months post-PAB due to progressive LV hypoplasia. |
